# Holobiont nitrogen control and its potential for eutrophication resistance in an obligate photosymbiotic jellyfish

**DOI:** 10.1186/s40168-021-01075-0

**Published:** 2021-06-02

**Authors:** Till Röthig, Giulia Puntin, Jane C. Y. Wong, Alfred Burian, Wendy McLeod, David M. Baker

**Affiliations:** 1 The Swire Institute of Marine Science and School of Biological Sciences, The University of Hong Kong, Hong Kong, Hong Kong, SAR of China; 2 Department of Bioresources, Fraunhofer Institute for Molecular Biology and Applied Ecology, Giessen, Germany; 3 Department of Biology, University of Konstanz, Konstanz, Germany; 4 Department of Animal Ecology & Systematics, Justus Liebig University, Giessen, Germany; 5 Marine Ecology Department, Lurio University, Nampula, Mozambique; 6 Department of Computational Landscape Ecology, UFZ– Helmholtz Centre for Environmental Research, Leipzig, Germany

**Keywords:** Stable isotope analysis, Tracer, Bacterial profiling, Environmental resilience, 16S rRNA gene

## Abstract

**Background:**

Marine holobionts depend on microbial members for health and nutrient cycling. This is particularly evident in cnidarian-algae symbioses that facilitate energy and nutrient acquisition. However, this partnership is highly sensitive to environmental change—including eutrophication—that causes dysbiosis and contributes to global coral reef decline. Yet, some holobionts exhibit resistance to dysbiosis in eutrophic environments, including the obligate photosymbiotic scyphomedusa *Cassiopea xamachana*.

**Methods:**

Our aim was to assess the mechanisms in *C. xamachana* that stabilize symbiotic relationships. We combined labelled bicarbonate (^13^C) and nitrate (^15^N) with metabarcoding approaches to evaluate nutrient cycling and microbial community composition in symbiotic and aposymbiotic medusae.

**Results:**

C-fixation and cycling by algal Symbiodiniaceae was essential for *C. xamachana* as even at high heterotrophic feeding rates aposymbiotic medusae continuously lost weight. Heterotrophically acquired C and N were readily shared among host and algae. This was in sharp contrast to nitrate assimilation by Symbiodiniaceae, which appeared to be strongly restricted. Instead, the bacterial microbiome seemed to play a major role in the holobiont’s DIN assimilation as uptake rates showed a significant positive relationship with phylogenetic diversity of medusa-associated bacteria. This is corroborated by inferred functional capacity that links the dominant bacterial taxa (~90 %) to nitrogen cycling. Observed bacterial community structure differed between apo- and symbiotic *C. xamachana* putatively highlighting enrichment of ammonium oxidizers and nitrite reducers and depletion of nitrogen-fixers in symbiotic medusae.

**Conclusion:**

Host, algal symbionts, and bacterial associates contribute to regulated nutrient assimilation and cycling in *C. xamachana.* We found that the bacterial microbiome of symbiotic medusae was seemingly structured to increase DIN removal and enforce algal N-limitation—a mechanism that would help to stabilize the host-algae relationship even under eutrophic conditions.

**Video abstract**

**Supplementary Information:**

The online version contains supplementary material available at 10.1186/s40168-021-01075-0.

## Background

Multicellular life depends on microorganisms for tight symbiosis, for their ability to drive biogeochemical processes, thus providing nutrients, or both [1]. Hosts and their associated microbial communities, together referred to as holobionts, have received increasing attention, especially in the context of ongoing global declines in coral reefs [2]. Cnidarian holobionts include bacteria, archaea, fungi, and viruses. Some form additional photosymbioses with endosymbiotic unicellular dinoflagellates from the family Symbiodiniaceae [3, 4]. The associated microorganisms, in particular bacteria and Symbiodiniaceae, are tightly linked to holobiont nutrient cycling and health [5].

The inclusion of algae allows the holobiont to directly access autotrophically fixed carbon (C) and thereby thrive in nutrient limited environments where heterotrophic food supply is strongly limited [6]. Photosynthetically fixed C is translocated mostly in the form of glucose from algae to host [7], but also other metabolites are exchanged [8]. In some cnidarian species (e.g. *Cassiopea* spp.), phototrophic C fixation can completely cover the host’s energetic requirements [9]. In turn, the host provides protection and favourable conditions in respect to light and CO_2_ [10]. This symbiosis is the foundation of coral reefs, which provide on a global level ecosystem services with an estimated value of US$375 billion per year [11].

The cnidarian-symbiodinian relationship includes facultative associations, for instance *Exaiptasia* and the stony coral *Astrangia poculata* and obligate association in most tropical reef-building corals. This symbiosis is remarkably sensitive to environmental changes and environmental stressors can cause its breakdown resulting in coral bleaching [12]. Such loss of photosynthetic pigments can be accompanied by the dysbiosis of other microbial associates [2, 13]. Obligate hosts may sometimes survive bleaching periods and recover [14], but nonetheless, climate change driven temperature rises and resulting mass bleaching have critical consequences for coral reefs on a global scale [15]. Consequently, key questions for conservation are what prevents host-algae dissociations and which factors support the reestablishment of disrupted symbiotic relationships once bleaching occurs.

Besides temperature, high nutrient levels can either directly cause bleaching or lead to dysbiosis, disease and host mortality [16]. Naturally, nitrogen (N) is limited in oligotrophic tropical coral reefs. It can be acquired by heterotrophic feeding of the host and passed on to its associates, e.g. as ammonium or amino acids, and further efficiently recycled within the holobiont [17]. N can also be directly obtained from seawater, where it is commonly present at low concentrations in the form of dissolved inorganic N (DIN, i.e. nitrate (NO_3_^-^), nitrite (NO_2_^−^) and ammonium (NH_4_^+^)), dissolved organic N, or particulate organic N [8]. Cnidarian hosts are able to incorporate ammonium but often associated microbial communities (particularly Symbiodiniaceae) account for most DIN uptake [17]. Symbiodiniaceae assimilate ammonium and nitrate, many microbial associates are involved in acquiring and efficiently (re)cycling N, and prokaryote diazotrophs can even fix atmospheric nitrogen [17–19]. The host benefits from a limited N availability [8, 20] as excess inorganic N can disrupt the host-symbiont partnership by altering the N:P (phosphorus) ratio within the holobiont and exacerbate heat-induced bleaching [17, 21]. Recently, prokaryote associates have also been linked to Symbiodiniaceae N limitation in coral holobionts by metabolizing and therefore limiting biologically available N [18, 22].

Cnidarian-symbiodinian holobionts are diverse and some are adapted to different trophic environments than coral reefs. Consequently, they vary in their heterotrophic feeding capacity and likely in their ability to assimilate and retain N [6, 23, 24]. A higher heterotrophic feeding capacity seems to correlate with an increased bleaching resistance in corals [23]. The mechanisms regulating nutrient acquisition and N cycling within the holobiont are still poorly resolved, but are key for understanding the intricate Cnidaria-Symbiodiniaceae symbiosis [17, 22, 25].

Of particular interest are organisms adapted to high nutrient environments as they might provide insights on how to effectively mitigate negative consequences of eutrophication. A potential mechanism of mitigation is the capacity of hosts to restrict N access from their symbionts. Examples of highly adapted organisms are obligate photosymbiotic jellyfishes of the genus *Cassiopea*, which persist in mangrove environments and seagrass beds with high nutrient loads as well as in more oligotrophic reef flats [9, 26, 27]. *C. xamachana* has recently gained increased attention as a suitable organism to study the cnidarian-symbiodinian relationship [28–30]. In contrast to most corals, its algal symbiosis can be discontinued for extended periods (>8 weeks), and re-infection with a range of Symbiodiniaceae species is easily possible [30, 31].

Consequently, *C. xamachana* provides a number of physiological characteristics turning it into a suitable model organism to study nutrient dynamics in Cnidaria and their resistance to nutrient-facilitated or induced bleaching. However, up to now comparatively little is known about its energy and nutrient budgets. Similar to other photosymbiotic scyphomedusae such as *Linuche unguiculata* [32], *C. xamachana* seems to attain the majority of its C requirements from photosynthesis, as bleached or light limited *C. xamachana* fail to maintain their mass even in the presence of high heterotrophic food concentrations [33, 34]. Further, adult medusae can utilize different DIN species including ammonium and nitrate [35] that are transferred between host and symbiont [36]. However, quantitative data on C and N acquisition is missing and it remains unknown in which form and at which rates elements are cycled among members of the holobiont in *C. xamachana*.

In this study our aim was to assess energy and nutrient cycling in *C. xamachana* to identify mechanisms stabilizing host-Symbiodiniaceae relationships in eutrophic environments. A key objective was to consider the microbiome due to its probable functional importance. Consequently, we combined pulse-chase experiments to trace isotopic labels with host physiological responses and gene amplicon sequencing of the bacterial microbiome. This allowed us to compare energy cycling in symbiotic and aposymbiotic individuals and relate nutrient uptake and turnover (or assimilation) rates with biodiversity and composition of the bacterial microbiome in *C. xamachana*.

## Material and methods

### Study organism and preparation of experiments

*Cassiopea xamachana* specimens employed in this study belong to strain T1A (draft genome available [37]). All individuals were from the same cohort, monoclonal, and propagated asexually as polyps. Associated Symbiodiniaceae were identified as clade A (the representative sequence matched A3 closely) by ITS2 sequencing (tested on 8 representative individuals, primers ‘ITS2symbF’: 5′ TGTGAATTGCAGAACTCCGT 3′ and 'ITS2symbR': 5′ TTTCCAAAGTCCTTTTCATCTTTC 3’ covering the full ITS2 and partial 5.8S and 28S genes, length: ~667 bp) and a subsequent blastn search (query cover: 100 %, Identity: 99 %). Adult medusae were maintained in a 500 L aquarium equipped with a filtration system, protein skimmer and reef sand. Two LED lights (Prime 16HD Reef, AquaIllumination) were set to a 12:12 h light to dark cycle at 100–150 μmol photons m^−2^ s^−1^. The medusae were kept in artificial seawater (ASW) with 35 PSS-78 salinity at 27–29°C temperature and fed ad libitum with freshly hatched or frozen *Artemia salina* 3–5 times a week.

For the experimental preparation, 35 adult symbiotic medusae (49 ± 7 mm bell diameter) were randomly selected and transferred to a separate tank (~15 L) containing filtered ASW (0.22 μm, changed daily) and air stones. Specimens were acclimated for 4 days. Aposymbiotic medusae were prepared by menthol-bleaching following a protocol modified from Matthews et al. [38]. Briefly, additional 20 individuals were incubated in ASW spiked with 0.38 mM menthol (from 1.28 M stock solution of 99 % menthol (Sigma-Aldrich) in 95 % ethanol) 8 h per day for 4 days. Incubation chambers were placed in a plant incubator (MLR-352, Panasonic) at 28°C and ~350 μmol photons m^−2^ s^−1^. Medusae were considered fully bleached when no fluorescence was detectable (Imaging-PAM, Waltz, Germany) and Symbiodiniaceae cells were not detected under a light microscope (Olympus Optical, mod. CHK at 400×). All 20 fully bleached medusae were kept in a separate, Symbiodiniaceae-free (0.22 μm ASW) system for 21 days at the same temperature and light regime as in the rearing tank.

### Experimental procedure—pulse-chase labelling experiment

**Fig. 1:**
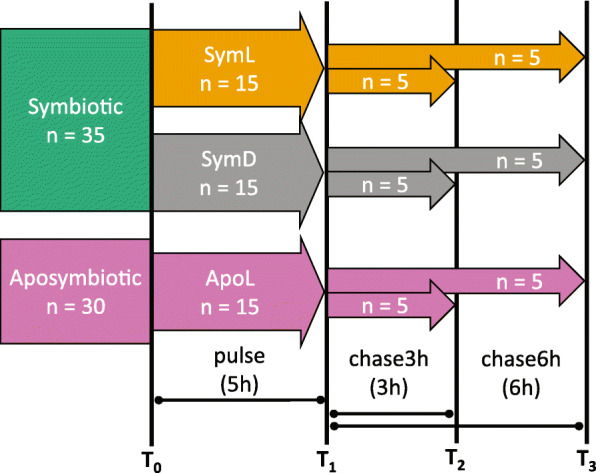
Experimental set-up. Boxes depict treatments (colour) and number of specimens (n). Treatments include symbiotic-light (SymL, orange), symbiotic-dark (SymD, grey), and aposymbiotic-light (ApoL, pink). Each vertical line represents a sampling event (T0, T1, T2, and T3) for 5 specimens from each treatment

In order to assess inorganic N and C assimilation, we performed a pulse-chase isotope labelling experiment with three treatments. Treatments included symbiotic individuals incubated under light conditions (SymL), symbiotic individuals incubated in darkness (SymD), and aposymbiotic individuals incubated in light (ApoL) (Fig. 1). Prior to the experiment, five symbiotic and five aposymbiotic specimens were sampled to establish natural stable isotope ratios (T0; Fig. 1). The remaining specimens (30 symbiotic and 15 aposymbiotic) were randomly assigned to one of the three treatments. The experiment consisted of an incubation with tracers (pulse), followed by a tracer-free incubation (chase). All incubations were performed in glass jars (‘incubation chamber’; IKEA Korken, ~1L) in incubators (MLR-352, Panasonic) at 28°C. Light was adjusted to 150 μmol photons m^−2^ s^−1^.

At the start of the experiment, each medusae was transferred to an incubation chamber filled with ^13^C and ^15^N enriched seawater (Sigma-Aldrich; 117 μM NaH^13^CO_3_, 98 atom % ^13^C; 1.18 mM Na^15^NO_3_, 98 atom % ^15^N). Dissolved oxygen (DO) was measured for each chamber (YSI® ProODO™ optical DO sensor, Yellow Springs, USA), then chambers were closed airtight without any remaining bubbles and randomly arranged in the incubators. After ~5 h, chambers were opened one by one and DO measured (Fig. 1). Each medusa was rinsed with filtered ASW and five specimens from each treatment (SymL, SymD, and ApoL; *n* = 15) were sampled (T1). Sampled individuals were measured (bell diameter using standard callipers), rinsed with abundant MilliQ water, wrapped in sterile aluminium foil, frozen at −80°C for about 30 min, and stored at −20°C until further processing. Meanwhile, the incubation chambers were carefully cleaned (bleach and MilliQ) and filled with filtered (tracer-free) ASW. The remaining medusae (*n* = 30) were transferred back into the chambers for the chase part of the experiment. Chambers were closed and handled as described above. After ~3 h, five individuals from each treatment (*n* = 15) were sampled (T2, ‘chase3h’), and the remaining individuals (*n* = 15) were sampled after another 3 h (T3, ‘chase6h’).

### Wet weight and bell diameter

During the experiment, only bell diameter (BD) was measured to minimize handling stress. In order to establish BD to wet weight (WW) relationships, we measured both in additional 35 symbiotic and 12 aposymbiotic individuals. We examined changes in mass and size of the 12 aposymbiotic medusae by BD and WW measurements 2, 8, and 25 days after the end of the menthol treatment (Additional File 1: Fig.S1). Measurements after 25 days were used to establish the BD-WW relationships for aposymbiotic individuals (the isotopic pulse-chase experiment was implemented 21 days after bleaching).

To measure WW, medusae were individually captured with a fine plastic net, gently shaken to remove excess water and weighted in a cup containing ASW. Immediately after weighing, maximum bell diameter (BD) was measured using standard callipers. WW-BD relationships were established using linear regressions and the best model was selected based on Akaike information criterion (AIC), resulting in a second-degree polynomial function for the symbiotic and a linear function for the aposymbiotic medusae (Additional File 1: Fig.S2).

### Productivity

DO concentrations before and after each incubation were used to calculate respiration (R; for dark incubation and for aposymbiotic medusae) and net primary production (P_n_; for light incubation of symbiotic specimens) rates as

1 $$ {P}_n\left[ mg{O}_2\ {g}^{-1}\ {h}^{-1}\right]=\frac{\Delta  {O}_2\left[ mg\ {L}^{-1}\right]\ast \left({V}_{\mathrm{chamber}}-{V}_{\mathrm{medusa}}\ \left[L\right]\right)}{T\left[h\right]\ast WW\left[g\right]} $$

where *V*_chamber_ and *V*_medusa_ stand for incubation chamber volume and medusa volume, respectively. *V*_medusa_ was calculated from WW based on the seawater density at 35 PSS-78, 28°C and standard atmospheric pressure. For symbiotic medusae, gross primary production (*P*_g_; *P*_g_ = *P*_n_+ *R*) and the *P*_g_:*R* ratio were calculated for the 5 h light pulse period. Of note, these values are approximations as there are systematic differences between light and dark respiration and both can vary over time [39].

### Heterotrophic nutrient dynamics

To assess the relative importance of heterotrophic feeding in *C. xamachana*, symbiotic specimens were fed with live zooplankton enriched with ^13^C and ^15^N. Zooplankton was enriched by first culturing *Isochrysis galbana* (haptophyte alga) for four days in a modified F/2 medium enriched with 750 mg L^−1^ NaH^**13**^CO_3_ (98 % heavy isotope) and 75 mg L^−1^ Na^15^NO_3_ (98 % heavy isotope, accounting for all nitrate present in the medium) to enrichment levels of ~10 AP^13^C (atom percent, see below) and ~15 AP^15^N. Isotopically enriched algae were fed to *Artemia salina* for 24–48 h. Four *C. xamachana* medusae (~3 cm BD) were starved for three days under normal light conditions, then fed ad libitum with labelled *A. salina* and sampled after ~5–6 h to allow for complete digestion (based on visual inspection of the gastric cavity). Each individual was thoroughly rinsed with ASW and MilliQ, and the gastric cavity excised with a clean scalpel to exclude partially undigested food particles. Medusae were then preserved as described above for stable isotope analyses (SIA).

### Sample processing

All frozen *C. xamachana* samples were homogenized. Aliquots were taken from all treatments for microbial analyses after isotopic labelling (pulse; *n* = 15). The tissue homogenate of each sample was separated by centrifugation into host and algal symbiont fractions. Algal cell counts were conducted using a haemocytometer under a light microscope. Algal and host fractions were freeze-dried before SIA. For detailed sample processing see Additional File 2: Text S1.

### Stable isotope analysis (SIA)

SIA was performed via combustion in a Eurovector EA3028 elemental analyser coupled to a Nu Instruments Perspective-series stable isotope ratio mass spectrometer in continuous flow mode. SIA results were firstly expressed in *δ*^15^N and *δ*^13^C, based on the isotopic composition of atmospheric N_2_ and on the Pee Dee Belemnite (PDB) standard, respectively. Acetanilide standards measurements were used to calculate the machine precision as percent relative standard deviation (standard deviation/mean × 100). Measurement precision in the pulse-chase experiment was 1.4 % for *δ*^13^C and 4.1 % for *δ*^15^N, while it was 0.2 % for *δ*^13^C and 12.8 % for *δ*^15^N for the heterotrophic experiment. For the pulse-chase experiment, each fraction (host and algae) from each medusa was measured in duplicates. The mean of both measurements was used for statistical analysis. Due to low sample mass for the algal symbiont fraction, only one reading was obtained in three samples (SymD-pulse, SymD-chase6h and SymL-chase6h). Isotope abundances were then converted to atom percent of ^13^C (AP^13^C) and ^15^N (AP^15^N) following Fry [40] after

2 $$ {AP}^HE=\left[\frac{{}^HE}{{}^LE{+}^HE}\right]\times 100 $$

where *H* and *L* refer to the number of light and heavy isotope atoms of the element *E* (i.e. C or N). Enrichment of both fractions in the heterotrophic experiment was calculated as atom percent excess (APE = AP_sample_ − AP_controls_). Then the enrichment of the symbiont was converted to percentage of enrichment relative to the host’s enrichment ((APE_Algal_ / APE_Host_) × 100). When necessary, a certified acetanilide standard was spiked in samples from the heterotrophic experiment to increase sample mass and/or to dilute highly enriched samples. APE values were then derived from mass balance equations.

### SIA data analysis

Data analysis was preformed after normality and homogeneity of variance were confirmed. Enrichment was tested with pairwise Welch two-sample upper-tailed *t*-tests applying Holm corrections for multiple comparisons. Effects of treatment, incubation and fraction were tested using linear mixed effect models or, when failing statistical requirements, with Kruskal-Wallis test by rank followed by post-hoc Wilcoxon rank sum exact test with Holm correction. The same non-parametric approach was used to test for difference in productivity (*P*_n_) and respiration (*R*) among treatments. All tests were performed in R version 4.0.2 [41].

### Bacterial community analysis

DNA from five medusae per treatment was extracted from homogenized tissue using a modified 2x CTAB chloroform protocol after Coffroth et al. [42]. DNA was quantified on a Multiskan GO (Thermo Fisher Scientific, Waltham, USA). The primers 784F (5′-TCGTCGGCAGCGTCAGATGTGTATAAGAGACAGAGGATTAGATACCCTGGTA-3′) and 1061R (5′-GTCTCGTGGGCTCGGAGATGTGTATAAGAGACAGCRRCACGAGCTGACGAC-3′) [43] with Illumina over-hang adaptor sequences (underlined above) were used to amplify the variable regions 5 and 6 of the 16S rRNA gene. For amplification and sequencing details see Additional File 2: Text S2.

Sequencing results were processed in Qiime2 [44]. Forward and reverse reads were split according to barcodes and assembled to contigs. Contigs >310 bp and those containing ambiguous bases were discarded. Sequences were quality filtered and categorized in amplicon sequence variants (ASVs) using DADA2 [45]. The resulting 303 ASVs were aligned against SILVA [46], release 138.1, using a primer-specific classifier trained in Qiime2. If present, chloroplasts, mitochondria, *Archaea*, eukaryotes and at the phylum level unknown sequences were removed. In total, we produced 16 16S rRNA gene libraries containing 221,216 sequences (252,674 before quality control) from one extraction control, five SymL, five SymD, and five ApoL *C. xamachana* samples. ASVs with an occurrence of ≥10 % in the extraction control compared to all samples were removed (i.e. 7 ASVs removed, 6 occurred only in the control; Additional File 3: Table S2) to account for potential contamination. Then stacked column plots depicting bacterial community compositions at the family level were constructed, and alpha diversity indices (ASV richness, Simpson and Shannon diversity) were calculated after implementation of a combined rarefaction-extrapolation procedure [47]. Evenness was computed from unrarefied data and phylogenetic diversity was established after Faith [48]. Alpha diversity indices were selected to provide largely independent measures (richness, evenness, and Faith) and comparability with other studies (Shannon and Simpson). Indices were compared among treatments using ANOVA analyses, or Kruskal-Wallis tests if assumptions of heteroscedasticity could not be met after data transformations. We used relative ASV densities (i.e. contribution of ASVs to total no. of reads per sample) computed from unrarefied data to establish a Bray-Curtis dissimilarity matrix and visualized sample similarity in a non-metric dimension scaling (NMDS) biplot using vegan [49]. Differences between samples and treatments were assessed using PERMANOVAs (Holm correction applied for multiple pairwise comparisons). Further, we assessed differences in within-treatment variability in community similarity, by pooling all pairwise Bray-Curtis similarity scores for within-treatment comparisons of samples and assessing differences among treatments using a Kruskal-Wallis test. All statistical tests have been performed in R and plots were constructed using ggplot2 [50]. The script can be found in Additional File 4.

Differences in predicted functional profiles based on phylogenetic inference of the associated bacterial communities were assessed using METAGENassist [51]. We used the cleaned ASV table (Additional File 3: Table S2) to perform ‘taxonomic-to-phenotypic mapping’ in METAGENassist, where all ASVs were taxonomically assigned and mapped, condensed into 184 functional taxa, and filtered based on interquartile range [52]. The remaining 175 functional taxa were normalized across samples by sum and over taxa by autoscaling. We analysed the dataset for ‘metabolism by phenotype’ using the Euclidean distance measure and clustered the 15 most differentially abundant metabolic processes (selected with random forest) using single algorithm.

## Results

### Bleaching and productivity

Menthol bleaching successfully produced aposymbiotic *C. xamachana* medusae after ~10 incubation days (Additional File 1: Fig.S1). However, even with regular feeding (≥ thrice a week ad libitum) aposymbiotic medusae shrank from 2.54 ± 0.47 to 1.35 ± 0.24 g over 29 days and survived for little over ten weeks. Symbiotic and aposymbiotic medusae differed in their WW to BD relationship (Additional File 1: Fig.S1, S2). Respiration (*R*) in aposymbiotic (49 ± 8 μg O_2_ g^−1^ h^−1^, measured under light conditions) was higher (*p*_*Holm*_ < 0.001) than in symbiotic (28 ± 14 μg O_2_ g^−1^ h^−1^, measured under dark conditions) medusae. Gross primary production (P_g_) for symbiotic specimens was 56 ± 15 μg O_2_ g^−1^ h^−1^ and the *P*_g_:*R* ratio for the 5 h pulse period was 2.0 ± 0.7.

### Inorganic carbon and nitrogen

**Fig. 2:**
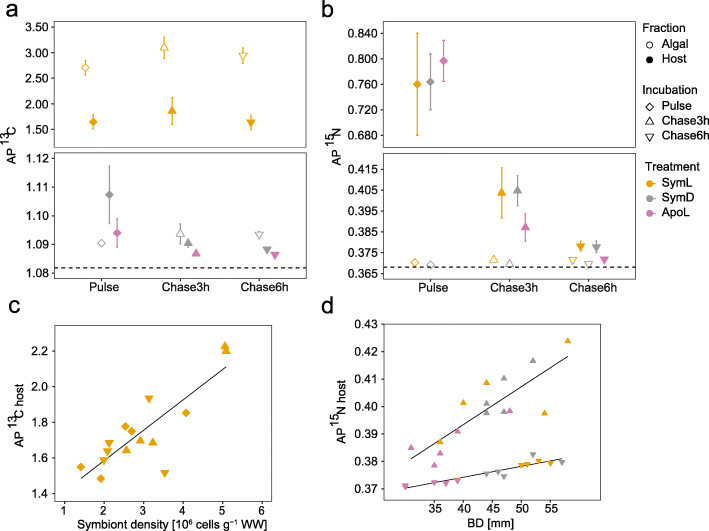
Nutrient dynamics in *C. xamachana*. Displayed are mean values (*n* = 5) per group for **a** C enrichment from NaH^13^CO_3_ expressed as AP^13^C and **b** N enrichment from Na^15^NO_3_ expressed as AP^15^N after the pulse (5 h), chase3h (3 h after pulse), and chase6h (6 h after pulse). Both panels are depicted with broken *y*-axes to facilitate comparisons between less enriched samples. Treatments included light-incubated symbiotic medusae (SymL), dark-incubated symbiotic medusae (SymD), and light-incubated aposymbiotic medusae (ApoL). Error bars represent 95 % confidence intervals and dashed line natural isotope ratios (*n* = 5; 1.081 AP^13^C and 0.368 AP^15^N). Further shown are **c** the regression between host C enrichment and symbiont density expressed as AP^13^C for SymL (*n* = 15; y = 1.24 + 0.17x; *r*^2^_Adj_ = 0.68, *p* < 0.001) and **d** the regressions between host ^15^N enrichment and medusae size expressed as AP^15^N from all treatments for chase3h (*y* = 0.3381 + 0.0014x; *r*^*2*^_*Adj*_ = 0.67, *p* < 0.001) and chase6h (*y* = 0.3583 + 0.0004x; *r*^*2*^_*Adj*_ = 0.86, *p* < 0.001; each *n* = 15)

All fractions in all treatments were significantly enriched in ^13^C and ^15^N after the pulse, chase3h and chase6h (for all *p*_*Holm*_ < 0.01) compared to the controls (Fig. 2a, b; Additional File 3: Table S3, S4). As anticipated, the algal fraction of the SymL treatment yielded the highest AP^13^C enrichment (2.57–3.35), followed by the respective host fraction (1.48–2.23). Owing to the lack of photosynthetic activity, both SymD and ApoL showed a much smaller overall enrichment (1.09–1.13). Further, we found that Symbiodiniaceae densities were an important factor explaining variation in ^13^C enrichment of the SymL host fraction (*r*^*2*^_*Adj*_ = 0.68, slope = 0.17, *p*_*lm*_ < 0.001, Fig. 2c). We also found a good accordance between host AP^13^C and net primary production (P_n_) computed from O_2_ measurements (*r*^*2*^_*Adj*_ = 0.74, slope = 10.84, *p*_*lm*_ < 0.001, Additional File 1: Fig.S3). Surprisingly, the highest ^13^C enrichment for both the host and algal fraction of SymL occurred at chase rather than at pulse (AP^13^C_Chase3h_ > AP^13^C_Pulse_ and AP^13^C_Chase3h_ > AP^13^C_Chase6h_, *p*_*LME*_ < 0.01). A similar delay in tissue enrichment was also found in the algal fraction of SymD, with the greatest enrichment at chase6h (*p*_*Wilcox*_ < 0.05). Conversely, SymD host fraction peaked at pulse and showed lowest enrichment levels at chase6h.

In contrast to ^13^C, ^15^N enrichment was always higher in the host than in the algal fraction. AP^15^N in the algal fraction of SymL was slightly higher than in SymD indicating a possible link between nitrate assimilation and photosynthesis (*p*_*LME*_ < 0.001). In the host fraction, ^15^N enrichment was highest at pulse (0.64–0.90) and dropped below 0.42 at chase for all treatments (Fig. 2b). At pulse, there were no significant differences between treatments (*p*_*ANOVA*_ > 0.05) (Additional File 3: Table S4), but ApoL samples experienced a larger drop in AP^15^N (i.e. N turnover rates) during the chase (*p*_*LME*_ < 0.01; Fig. 2b). Notably, AP^15^N levels correlated significantly with medusa size for both chases when considering the host fractions from all treatments (*r*^*2*^_*Adj* =_ 0.67 and 0.86 respectively, *p*_*lm*_ < 0.001 for both; Fig. 2d). However, the effect of medusa size seemed to be solely related to N turnover rates as there was no relationship between BD and AP^15^N after the pulse experiment (*r*^*2*^_*Adj*_ = 0.04, *p*_*lm*_ > 0.05). AP^15^N values of the algal fraction in symbiotic individuals from both treatments peaked at the end of the chase (AP^15^N_chase6h_ > AP^15^N_pulse_ and AP^15^N_chase6h_ > AP^15^N_chase3h_; *p*_*LME* <_ 0.001), in contrast to the pattern observed in the host fraction.

### Organic carbon and nitrogen

Medusae were fed ^13^C and ^15^N labelled *A. salina* (Additional File 3: Table S5) to assess the cycling of heterotrophic nutrients in *C. xamachana*. Both the symbiont and the host fraction were enriched (APE host fraction > 1.19 and > 0.46, APE algal fraction > 1.12 and > 0.43 for ^13^C and ^15^N, respectively) and as expected prey enrichment level affected jellyfish enrichment (Additional File 3: Table S3). As direct consumer of the prey, the host showed higher enrichment levels than the algal fraction. Nevertheless, algal enrichment reached 36.7 ± 4.6 % of the host ^13^C and 70.3 ± 4.0 % of the host ^15^N enrichment highlighting fast and extensive elemental cycling.

### Bacterial communities associated with *C. xamachana*

**Fig. 3:**
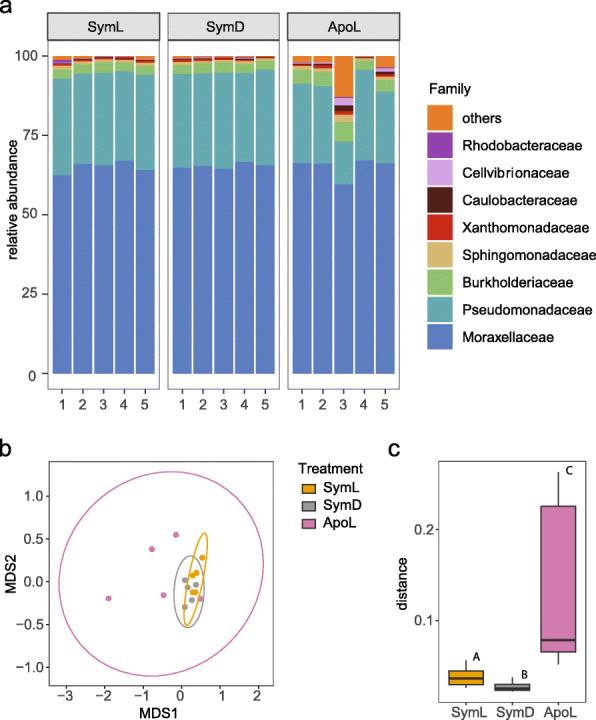
*C. xamachana* associated bacterial communities. **a** Stacked column plot representing the bacterial community composition associated with *C. xamachana* on the family level (SILVA database). Each colour represents one of the eight most abundant families; all 85 other families are grouped as ‘others’. **b** Clustering of *C. xamachana* samples based on Bray-Curtis dissimilarity of bacterial community abundances in a non-metric multi-dimensional scaling plot (NMDS; stress value < 0.078). Circles indicate 95 % confidence ranges. **c** Boxplots of pairwise Bray-Curtis dissimilarities of samples within treatments. Statistical comparisons are based on Kruskal-Wallis rank sum test (chi^2^ = 21.5, df = 2, *p* < 0.001). Letters indicate significant differences of pairwise comparisons (*p*_*Holm*_ < 0.01). Treatment abbreviations are same as in Fig. 2

The bacterial microbiome of *C. xamachana* was dominated by *Moraxellaceae* (>60 %) and *Pseudomonadaceae* (~15-25 %) across all samples (Fig. 3a). Differences between samples were generally subtle, but ApoL3 contained a higher abundance of ‘others’, i.e. families with a comparably low abundance.

Further, we assessed differences in the bacterial community structure among treatments by computing pairwise sample similarity (Bray-Curtis index) and displaying them in an nMDS biplot (Fig. 3b). ApoL samples showed slight differences to non-bleached treatments, which were significant (ApoL and SymL; *p*_PERMANOVA_ = 0.036) or marginally non-significant (ApoL and SymD; *p*_PERMANOVA_ = 0.068). A key difference, however, was within treatment heterogeneity in community composition (Fig. 3b, c). A comparison of within group community similarity across treatments revealed a much higher compositional heterogeneity in ApoL compared to the two treatments with symbiotic algae (Kruskal-Wallis Test; chi2= 21.463, df = 2, *p*_Wilcoxon_ < 0.01). On average, the number of reads, richness, Shannon and Simpson diversity, phylogenetic diversity (Faith), and evenness were lower in both symbiotic groups than in the aposymbiotic samples, which showed a markedly higher variability (i.e. standard deviation) for all measures (Table 1).

**Table 1:** Summary statistics detailing bacterial communities associated with *C. xamachana*

	# of reads	Richness	Shannon	Simpson	Faith	Evenness
SymLPul1	12437	38	3.76	2.39	0.333	0.364
SymLPul2	13394	39	3.49	2.26	0.367	0.341
SymLPul3	11826	27	3.43	2.26	0.184	0.374
SymLPul4	16425	29	3.27	2.17	0.206	0.352
SymLPul5	13997	39	3.51	2.29	0.255	0.343
**Mean**	**13616**	**34**	**3.49**	**2.27**	**0.269**	**0.355**
**STDV**	**1780**	**6**	**0.18**	**0.08**	**0.079**	**0.014**
SymDPul1	13346	37	3.45	2.26	0.255	0.342
SymDPul2	17415	42	3.45	2.24	0.260	0.331
SymDPul3	13342	32	3.41	2.29	0.208	0.353
SymDPul4	14175	37	3.46	2.22	0.224	0.343
SymDPul5	15073	28	3.26	2.22	0.174	0.354
**Mean**	**14670**	**35**	**3.40**	**2.24**	**0.224**	**0.345**
**STDV**	**1693**	**5**	**0.08**	**0.03**	**0.036**	**0.010**
ApoLPul1	15829	53	3.95	2.25	0.336	0.345
ApoLPul2	17442	58	4.09	2.26	0.292	0.347
ApoLPul3	14280	140	10.23	3.02	0.618	0.470
ApoLPul4	12120	25	3.12	2.14	0.267	0.353
ApoLPul5	19938	69	4.45	2.26	0.358	0.352
**Mean**	**15922**	**69**	**5.17**	**2.39**	**0.374**	**0.373**
**STDV**	**2983**	**43**	**2.87**	**0.36**	**0.141**	**0.054**

Treatments: *Apo* aposymbiotic, *Sym* symbiotic, *L* light, *D* dark, *Pul* sampled after pulse incubation

Moreover, we assessed ASVs based on their ubiquity as a potential indicator of their functional importance [53]. The core microbiome, i.e. the ASVs present in all 15 medusae, consisted of 10 ASVs, including 9 of the 10 most abundant taxa (Additional File 3: Table S2). The symbiome (ASVs occurring in all SymL and SymD samples; of note, the respective ASVs may be members of multiple ‘biomes’) consisted of 12 ASVs (*Acinetobacter* sp., *Ruegeria* sp., and the 10 core microbiome members). The apobiome also consisted of 12 ASVs (*Cellvibrio* sp., *Ralstonia* sp., and the 10 core microbiome members) (Additional File 3: Table S2).

### Taxonomy-based functional profiling of bacterial communities in *C. xamachana*

**Fig. 4:**
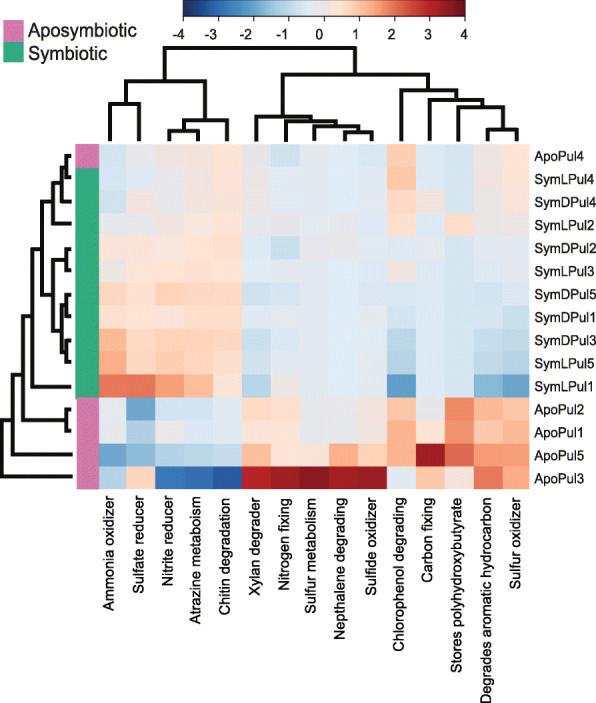
Predicted taxonomy-based functional differences of bacterial communities in *C. xamachana.* Enrichment is depicted in red and depletion in blue on a relative scale. Treatment abbreviations are same as in Fig. 2

Based on a METAGENassist analysis, we identified the 15 functional categories, which showed the largest differences across all bacterial microbiome samples (Fig. 4). Further, we used these categories to cluster all samples, which revealed a clear differentiation between aposymbiotic and symbiotic samples. Only ApoLPul4, the aposymbiotic sample with the lowest alpha diversity (expect evenness) (Table 1), was clustering more closely with the symbiotic than with the aposymbiotic samples. Differences included in particular a number of functions related to N metabolism. Functional traits that would limit inorganic N availability for the symbiotic algae, such as ammonium oxidizers and nitrite reducer were enriched in symbiotic microbiomes. On the other hand, nitrogen fixing, which potentially increases the nitrogen availability for the holobiont was depleted in symbiotic medusae. Further, an enrichment of sulphur oxidizers and a depletion of carbon fixers indicated lower O_2_ levels for microbiomes of symbiotic medusae (Fig. 4), which are required for effective N removal. Other functions like ‘degrades aromatic hydrocarbon’ and ‘xylan degraders’ were also more enriched in aposymbiotic sampled compared to ‘atrazine metabolism’ and ‘chitin degraders’ that were generally more depleted in these samples.

## Discussion

In this study, we assessed energy and nutrient dynamics in the *Cassiopea xamachana* holobiont to identify mechanisms that facilitate the maintenance of cnidarian-symbiodinian relationships under high nutrient conditions. The ability of *C. xamachana* to survive prolonged periods in an aposymbiotic state permitted us to disentangle the role of host and symbionts in nutrient dynamics, highlighting the value of *C. xamachana* as a model organism. Our results demonstrate that both autotrophically fixed and heterotrophically acquired C quickly cycled within the holobiont and was incorporated in both host and Symbiodiniaceae tissue*.* Nevertheless, heterotrophic feeding alone was not sufficient to cover the host’s needs and led to consistent weight loss. N assimilated from zooplankton prey was likewise shared among host and the algae. In contrast, inorganic N present in the surrounding water was effectively blocked off from Symbiodiniaceae. An analysis of host associated bacterial communities indicated that bacterial processes involved in N removal through ammonium oxidation and nitrite reductions were presumably enriched in the symbiotic hosts. Restricting the transmission of ambient DIN to Symbiodiniaceae can stabilize host-algal relationships in nutrient-rich environments and our data suggests that *Cassiopea xamachana*’s bacterial microbiome might play an important role in this process.

### The fate of carbon

The daytime photosynthesis respiration ratio (*P*_g_:*R* = 2) indicated that C fixation rates are sufficient to cover respiration even though they are lower—possibly due to handling stress—than previous measurements (2.04 for 24 h, including 12 h dark) [35]. As anticipated, light conditions facilitated the highest AP^13^C enrichment in Symbiodiniaceae and the host. Here, a large fraction of C was immediately transferred to the host (during the 5 h pulse), which is consistent with findings from coral holobionts [54]. Both host and symbiodinians maintained high ^13^C levels for at least 6 h, indicating an incorporation of C into cell tissues. In symbiotic holobionts, C transfer to the algae can be realized through respiration or in the form of organic C. Dissolved ^13^CO_2_ could diffuse into the host tissue and contribute to a ‘delayed enrichment’ (i.e. highest AP^13^C values after the 3 h chase) in our inorganic labelling experiment. Such dissolved CO_2_ pools utilized for photosynthesis were likely lost during sample preparation resulting in lower AP^13^C values in pulse samples.

In treatments lacking photosynthesis (SymD and ApoL treatments), ^13^C labelling also resulted in C assimilation, albeit at much lower rates (Fig. 2a). Baker et al. [24] explained host C enrichment in the dark by anaplerotic reactions that form intermediates of metabolic pathways including lipid synthesis and oxidation which could be performed by host and/or microbiome [55]. In the octocoral species assessed by Baker et al., however, the algal symbionts were not enriched after dark incubation. In *C. xamachana*, Symbiodiniaceae continued to accumulate C in the dark even after the pulse suggesting it originated from other holobiont members, in particular the host. Such C transfer from host to algae was also apparent from labelled *Artemia* and has been shown in coral from heterotrophic food sources [56, 57]. When bleached, the aposymbiotic holobiont enters a starvation mode that is characterized by a collapse in DIC uptake and cycling. Overall, C assimilation and (re)cycling was only effective in symbiotic *C. xamachana* holobionts under light conditions.

### The fate of nitrogen

The uptake of inorganic N (^15^NO_3_^−^) showed distinctly different patterns from C assimilation. DIN uptake rates were much lower compared to DIC and we found a continuous AP^15^N decline in the host after the pulse for all treatments. All host samples (independent of state and treatment) showed a similarly strong initial enrichment after the pulse. Interestingly, this enrichment was followed by a pronounced drop in AP^15^N in all treatments, which correlated with medusa size and hence surface-to-volume ratio (i.e. larger medusae maintained higher AP^15^N level during both chases; Fig. 2d). Freeman et al. [36] found a similarly quick increase and depletion of ^15^N in *C. xamachana* incubated with ^15^NO_3_^−^ and speculated that microbial activity could drive such high turn-over rates. The bacterial communities characterized here include abundant putative nitrogen cyclers (comprising nitrate reducers) that could contribute to the observed AP^15^N dynamics (see below). This pattern stands in stark contrast to coral host tissues, where ^15^N did not decrease over 14 days in a similar pulse-chase experiment [54], which indicates that corals are highly effective in N retention [17]. We argue that initial enrichment and subsequent size-correlated depletion in AP^15^N indicate a passive, concentration gradient driven movement of nitrate in and out of the host tissue that might work in concert with the associated prokaryotic community.

Importantly, the algal symbionts were unable to capitalize on available DIN indicating an effective control mechanism. N restriction as a mechanism of symbiont control has been suggested in coral and *Exaiptasia* as it is thought to maintain algal cell densities and ensure high rates of photosynthate translocation [20]. In corals, Tanaka et al. [54] found AP^15^N in the associated algae to be 4.7 times higher than in their hosts which highlights that only the algae are able to assimilate nitrate. In *C. xamachana,* however, the algal partners enrich slowly throughout the experiment, largely independent of the hosts ^15^N level. Overall low nitrate assimilation is in line with other ‘nutrient resistant’ cnidarian-symbiodinian associations. *L. unguiculata* [58] and *Exaiptasia pulchella* [59] removed none or only minimal nitrate from their surrounding water and *δ*^15^N enrichment from nitrate was not detectable in *Exaiptasia* [60] (notably, all of these are non-calcifying hosts and *Exaiptasia* spp. are not obligatory photosymbiotic). In other photosymbioses adapted to high nutrient concentrations, for instance *Hydra viridissima* [61] or *Paramecium bursaria* [62], some *Chlorella* endosymbionts have lost their assimilatory nitrate reduction pathway as an adaptation to symbiotic conditions. Such inability for nitrate uptake remains to be tested for *C. xamachana’s* algal partners. However, the host’s capacity to associate with several Symbiodiniaceae strains originating from ‘nutrient sensitive’ corals [30] argues for alternative mechanisms. Studies on such calcifying corals demonstrate that the photosymbionts were able to effectively assimilate nitrate and translocate it to their hosts in *Acropora pulchra* [63], *A. tenuis* [64], *Orbicella faveolata* [65], *Porites cylindrica* [54], and *Stylophora pistillata* [66]. Compared to *C. xamachana*, in a similar experimental setup (at similar C assimilation rates), the algal fraction of *O. faveolata* showed ^15^N enrichment two orders of magnitude greater (0.784 APE^15^N compared to 0.002 APE^15^N) and was remarkably higher than the host fraction enrichment [65]*.* In *C. xamachana*, Symbiodiniaceae are located in a symbiosome, a membrane complex wrapping the algal cells, which are predominantly situated in amoebocytes. While little is known about the function of (likely mobile) amoebocytes [31], the symbiosome is actively involved in nutrient transfer in *Exaiptasia pallida* and *Acropora digitifera* [67] and could be involved in restricting N access of the photosymbionts.

Unlike nitrate, organic ^15^N from labelled *Artemia* appeared freely transferable within *C. xamachana,* indicating that tight supply regulation might be specific for certain chemical forms. Heterotrophic or organic N, in contrast to nitrate, tends to improve health and metabolism in coral and may not harm the cnidarian-symbiodinian relationship [68, 69]. Ammonium, a metabolic waste product of the host, can also stimulate photosynthesis and carbon translocation to the host even under environmental stress and, importantly, as the preferred N source inhibit nitrate uptake in Symbiodiniaceae [66, 70]. By restricting access of external DIN for the algal symbionts and exerting control on the transfer of organic N and ammonium, the host presumably improves the holobionts (nutrient) resilience, which might be supported by the prokaryotic community (see below). Taken together, N cycling within the *C. xamachana* holobiont suggests an effective internal nitrate restriction of its algal associates, which might contribute to the high nutrient tolerance in *C. xamachana*.

### The associated bacterial microbiome shares characteristics with other cnidarians

Prokaryotic associates contribute to nutrient cycling and interact directly with host and Symbiodiniaceae. Data on the associated bacterial communities (and disease) in *C. xamachana* are lacking. Other, asymbiotic scyphozoans host largely species-specific bacterial communities that differ from the environment, across body parts and life stages, and are involved in asexual reproduction, health, and fitness of the host [71–74].

The bacterial communities associated with *C. xamachana* were dominated by two ASVs (*Acinetobacter* sp. and *Pseudomonas* sp.) which made up 85 ± 2 % of all sequences in all samples but ApoLPul3 (66 %). These and other abundant taxa have been found to be associated with cnidarians before (e.g. *Acinetobacter* [75], *Pseudomonas* [76–78], *Massilia* [79], *Sphingobium* [80]). However, employing a sequence-based blastn search of the eleven most abundant ASVs did not yield associations with marine hosts. As to date most published data are OTU-based, we employed this approach for comparison with other cnidarian bacterial microbiomes (Additional File 2: Text S2). *C. xamachana* samples contained between 51 and 300 distinct OTUs which is in the same range (~100) as the cnidarian anemones *E. pallida* [78] and *Hydra vulgaris* [81] (Additional File 3: Table S6). In coral, the numbers are more variable depending on species and environmental conditions, but are generally in the order of tens to hundreds of OTUs [76, 82, 83]. Considering the best blastn hits for each OTU, we found that 9 of the 18 core microbiome member OTUs have previously been identified in corals and 5 in *E. pallida* (Additional File 3: Table S7). This highlights similarities in cnidarian holobiont and points towards bacterial taxa that are potentially conserved within the phylum.

Aposymbiotic samples seemed to host more variable bacterial communities illustrated by larger variations in the alpha diversity indices and a significantly larger within-treatment dissimilarity compared to both symbiotic groups (Table 1, Fig. 3c). Stressed coral tend to display higher community dissimilarity [84], though that is not always the case [85]. Stress may leave them more vulnerable to invasion and thus associations to otherwise untypical residents, which may increase alpha diversity [86]. In this study, a lack of nutrients in the aposymbiotic medusae could have similar effects. In particular, a lack of the obligate photosymbionts and therefore the main energy source might cause an unbalance of the associated microorganisms. Members of the symbiome may also be linked to the cnidarian-symbiodinian association as suggested for *E. pallida* [78]. Interestingly, the symbiome we identified comprised 12 members, which were also present in most aposymbiotic samples. This is surprising as Symbiodiniaceae are thought to maintain core microbiomes of their own [87], even *in hospite* [18, 88]. In this context, *C. xamachana* provides a system to readily test Symbiodiniaceae*-Bacteria* associations in an obligate symbiosis as the host can be infected with different algal species [30].

### The role of the bacterial microbiome in holobiont nutrient cycling

The Symbiodiniaceae*-Bacteria* interactions have been hypothesized to be a hidden key for coral reef resilience [18]. Our data indicates that the majority of *C. xamachana*-associated *Bacteria* could be directly involved in nitrogen cycling and in particular denitrification. Based on a literature search, the 20 most abundant ASVs (Additional File 3, Table S2) belong to genera that can be linked to denitrification processes including *Acinetobacter* [89], *Pseudomonas* [90], *Massilia* [91], *Sphingobium* [92], *Stenotrophomonas* [93], *Cellvibrio* [94], *Brevundimonas* [95], Sphingomonas [96], *Ralstonia* [97], *Cutibacterium* (KEGG pathway *Cutibacterium acnes* KPA171202 [98]), *Flavobacterium* [99], and *Ruegeria* [100]. Further, *Acinetobacter* [89], *Pseudomonas* [90], Massilia [101], *Sphingomonas* [102], and *Flavobacterium* [99] have also been associated with nitrification processes and nitrifying bacteria have previously been linked to *Cassiopea* sp. [35]. These findings were supported by a sequence-based NCBI blastn search to assess putative functions. For the 20 most abundant ASVs, we identified 12 complete genomes at 100 % similarity to their respective amplicons, 6 at >99 %, and 2 at >97 %. All complete reference genomes included gene homologs presumably involved in N-cycling (Additional File 3: Table S8). Considering only genomes with 100 % match, 10 taxa contained at least one gene homolog potentially linked to denitrification. We further identified several gene homologs for nitrogen transport, ammonification, nitrification, and nitrogen fixation (Additional File 3: Table S8). Amplicon sequencing data are not quantitative and—like inferred functionality—should be interpreted with caution. However, the dominance of taxa presumably involved in nitrogen cycling and particularly denitrification is considerable. Both processes could be further supported by archaeal communities that were not assessed in this study [103]. While the pathways potentially involved remain to be elucidated (particularly considering oxygen sensitivity of nitrogenase; but see [104]), a combination of denitrifying, nitrifying, and ammonifying communities could effectively remove bioavailable N from the holobiont and support algal N limitation [22, 103, 105]. Future studies should target specifically functional genes to confirm and quantify N cycling processes (e.g. nitrite reductase *nir*, nitric oxide reductase *nor*, ammonia monooxygenase *amo*, and dinitrogenase reductase *nif*).

**Fig. 5:**
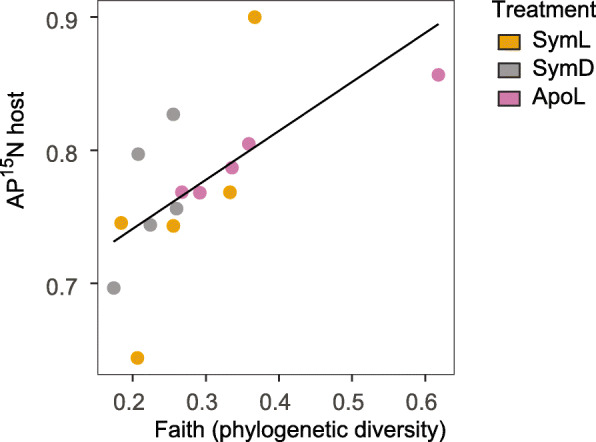
Bacterial phylogenetic diversity affects host N assimilation. Regression: *y* = 0.667 + 0.368 *x*, *r*^2^_*Ad*j_ = 0.39, *p* < 0.01. *n* = 15, all sampled after pulse incubation. Treatment abbreviations are the same as in Fig. 2

The associated bacterial communities further seem to contribute to holobiont N assimilation. In our study, ^15^N enrichment in the host tissue correlated positively with phylogenetic diversity of the associated bacterial community (Fig. 5). Of note, host (and algal) fraction measurements may include ^15^N that was assimilated by their associated prokaryotic community. However, based on the quick release of ^15^N after the chase (Fig. 2b), the holobionts’ overall assimilation of DIN is low and points towards a contribution of the microbial community. This positive relationship between bacterial phylogenetic diversity and ^15^N uptake rates fits well with the biodiversity-ecosystem functioning theory [106]. It has been shown that higher species numbers and phylogenetic diversity can enhance nutrient uptake and storage on an ecosystem level [107]. The data presented here hint that such a relationship might also exist within the holobiont/metaorganism concept.

Holobiont nutrient cycling changed fundamentally upon the cnidarian-symbiodinian dissociation after which the holobiont enters a starvation mode. The composition of associated bacteria also reflected the underlying physiological changes in medusa holobionts. Differences in the predicted functional profiles indicated that the bacterial communities changed from a nitrogen (‘ammonia oxidizer’, nitrite' reducer’, ‘sulphate reducer’) towards a sulphur (‘sulphur oxidizer’, ‘sulphide oxidizer’, ‘sulphur metabolism’)-based metabolism. This trend contrasts with findings on *Exaiptasia* where symbiotic polyps indicated higher sulphur metabolic activities, which was suggested to originate from Symbiodiniaceae derived dimethylsulphoniopropionate (DMSP) [78]. DMSP, however, is also present in (symbiotic) *C. andromeda* [108]. The enrichment of ‘ammonia oxidizer’ and ‘nitrite reducer’ in symbiotic *C. xamachana* indicates that biologically available N could be removed by the respective bacterial taxa supporting the algae’s nutrient limitation. This also contrasts findings in *Exaiptasia* where ‘nitrite reducer’ enriched in aposymbiotic samples [78]. While the predicted functional profiles corroborate nutrient deprivation in the medusae, the results should be interpreted with caution, as bacterial taxa putatively involved in N cycling were also dominant in aposymbiotic individuals (see above) and functional predictions remain putative. Future work employing metagenomics and metatranscriptomics may provide a more direct assessment of this relationship and the role of each holobiont member.

## Conclusions

Our data indicate that different holobiont members contribute to nutrient cycling in *C. xamachana*. The host assimilates organic C and N and transfers both to its algal associates, which are restricted in DIN access. The obligate symbiosis with Symbiodiniaceae provides access to autotrophically fixed C that might contribute to energetically costly DIN assimilation. The cnidarian-symbiodinian association further facilitates effective C and N cycling. The associated bacterial communities seem to contribute to DIN uptake, which is correlated with their phylogenetic diversity and the host to DIN turnover (correlated with size). Nitrate levels in the host and algal partner indicate an internal strategy to limit algal N access that differs from other cnidarians (especially coral) and might help to explain the high nutrient tolerance peculiar to *Cassiopea* spp. While ^15^N stemming from DIN can quickly enrich and deplete in the host tissue, the photosymbionts’ access is limited. Nitrate restriction could be realized at the symbiosome, which should be addressed in future research. Additionally, the holobiont hosts a remarkable abundance of putatively N-cycling related (and denitrifying) bacteria that presumably remove N from the host tissue. The combination of host DIN control and microbial DIN removal might enable the jellyfish to thrive in nutrient rich waters in contrast to coral. Recent ‘coral probiotics’ approaches could test whether these bacteria may also increase coral resilience in eutrophied habitats [70].

## Supplementary Information

**Additional file 1: Figure S1**. Effects of menthol bleaching on *C. xamachana.* (**a**) Photosynthetic efficiency throughout menthol exposure (day 1-4) and bleaching process. *n* = 12; Imaging-PAM (Walz, Germany); *F*_*o*_: dark-adapted minimal fluorescence yields; *F*_*m*_: dark-adapted maximal fluorescence yield; *F*_*v*_*/F*_*m*_: maximum quantum yield. (**b**) Response of bell diameter (BD) and wet weight (WW) to bleaching (mean, *n* = 12). (**c**) Visualization of photosynthetic efficiency (i.e. *F*_*o*_, *F*_*m*_, and *F*_*v*_*/F*_*m*_). **Figure S2.** Relationship between wet weight (WW) and bell diameter (BD) in (**a**) symbiotic (*n* = 35) [WW_symbiotic_ = 8.540 -0.494*BD + 0.009*BD^2^] and (**b**) aposymbiotic *C. xamachana* (*n* = 12) [WW_aposymbiotic_ = -1.440 + 0.100*BD]. Different colours indicate the number of days after start of the four-day menthol bleaching. Dashed vertical lines indicate minimum and maximum BD of medusae employed in the pulse-chase experiment. **Figure S3.** Net primary production (P_n_) and host carbon enrichment. Host fraction enrichment expressed as AP^13^C light-incubated medusae (SymL) from all incubations (pulse, chase 3h and chase 6h) (*n* = 15).

**Additional file 2: Text S1.** Extended Material and Methods. **Text S2.** OTU based bacterial analysis.

**Additional file 3: Table S2.** ASV abundance table detailing ASV numbers over samples, including taxonomy and representative sequences. Number of ASVs 303, of which 7 are contaminants (marked with *), core microbiome members in bold, 'symbiome' members underlined for symbiotic samples and 'apobiome' members underlined for aposymbiotic samples. **Table S3.** Detailed stable isotope measurements of *Cassiopea xamachana* and its associated Symbiodiniaceae in symbiotic-light (SymL), symbiotic-dark (SymD), aposymbiotic-light (ApoL) treatments and upon heterotrophic feeding. **Table S4.** Stable isotope analysis enrichment statistics. **Table S5.** Stable isotope labelling of *Isochrysis galbana* and *Artemia salina*. ^13^C and ^15^N enriched *Isochrysis galbana* algae were employed to label *Artemia salina* as a heterotrophic food source for *C. xamachana*. **Table S6.** OTU abundance table detailing OTU numbers over samples, including taxonomy and representative sequences. Number of OTUs 697, of which 9 are contaminants (marked with *), core microbiome members in bold, 'symbiome' members underlined for symbiotic samples and 'apobiome' members underlined for aposymbiotic samples. **Table S7.** Closest blastn hits considered for presumably important OTUs (including core microbiome, symbiome, and apobiome; assessed June 2020). **Table S8.** Closest complete genomes of the 20 most abundant ASVs based on an NCBI blastn search (assessed November 2020). Considered gene homologs are related to nitrogen cycling and transport.

**Additional file 4: R-scripts** employed to analyse the bacterial microbiome.

## Data Availability

Sequences determined in this study have been deposited in the NCBI Sequence Read Archive under accession no. PRJNA627421 (https://www.ncbi.nlm.nih.gov/bioproject/627421).
