# Supplementary material for: Holobiont nitrogen control and its potential for eutrophication resistance in an obligate photosymbiotic jellyfish
Source: Microbiome. 2021 Jun 2;9:127. doi: 10.1186/s40168-021-01075-0 (PMC8173792; doi:10.1186/s40168-021-01075-0)
Supplement: Supplementary file 2 — Additional file 1: Figure S1. Effects of menthol bleaching on C. xamachana. (a) Photosynthetic efficiency throughout menthol exposure (day 1-4) and bleaching process. n = 12; Imaging-PAM (Walz, Germany); Fo: dark-adapted minimal fluorescence yields; Fm: dark-adapted maximal fluorescence yield; Fv/Fm: maximum quantum yield. (b) Response of bell diameter (BD) and wet weight (WW) to bleaching (mean, n = 12). (c) Visualization of photosynthetic efficiency (i.e. Fo, Fm, and Fv/Fm). Figure S2. Relationship between wet weight (WW) and bell diameter (BD) in (a) symbiotic (n = 35) [WWsymbiotic = 8.540 -0.494*BD + 0.009*BD2] and (b) aposymbiotic C. xamachana (n = 12) [WWaposymbiotic = -1.440 + 0.100*BD]. Different colours indicate the number of days after start of the four-day menthol bleaching. Dashed vertical lines indicate minimum and maximum BD of medusae employed in the pulse-chase experiment. Figure S3. Net primary production (Pn) and host carbon enrichment. Host fraction enrichment expressed as AP13C light-incubated medusae (SymL) from all incubations (pulse, chase 3h and chase 6h) (n = 15). [file 40168_2021_1075_MOESM2_ESM.docx]

**Holobiont nitrogen control and its potential for eutrophication resistance in an obligate photosymbiotic jellyfish**

Till Röthig^1,2,3,#,*^, Giulia Puntin^1,4,#^, Jane CY Wong^1^, Alfred Burian^5,6^, Wendy McLeod^1^, David M Baker^1,*^

^1^ The Swire Institute of Marine Science and School of Biological Sciences, The University of Hong Kong, Hong Kong, SAR China

^2^ Department of Bioresources, Fraunhofer Institute for Molecular Biology and Applied Ecology, Giessen, Germany

^3^ Department of Biology, University of Konstanz, Konstanz, Germany

^4^ Department of Animal Ecology & Systematics, Justus Liebig University, Giessen, Germany

^5^ Marine Ecology Department, Lurio University, Nampula, Mozambique

^6^ Department of Computational Landscape Ecology, UFZ– Helmholtz Centre for Environmental Research, Leipzig, Germany

# contributed equally

* Corresponding author

**Additional Information File 1 – Supplementary Figures**


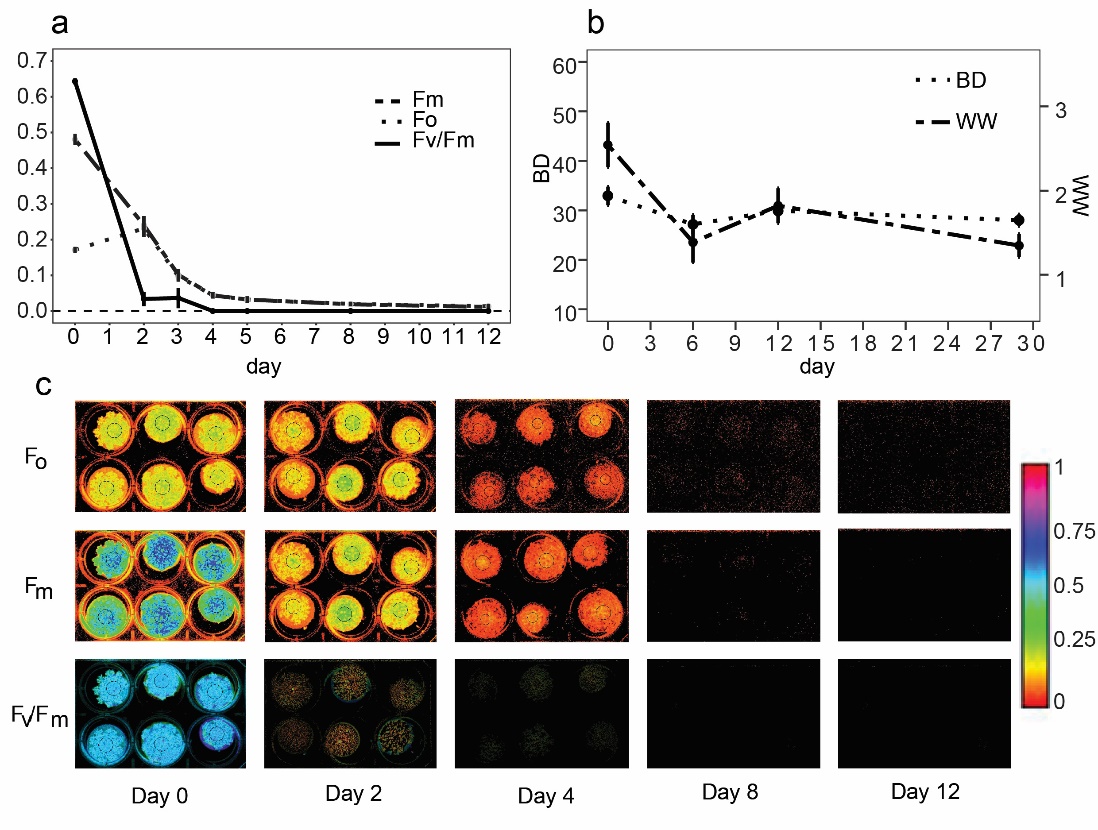


**Fig.S1. Effects of menthol bleaching on *C. xamachana****.* (**a**) Photosynthetic efficiency throughout menthol exposure (day 1-4) and bleaching process. n = 12; Imaging-PAM, Walz, Germany; *F_o_*: dark-adapted minimal fluorescence yields; *F_m_*: dark-adapted maximal fluorescence yield; *F_v_/F_m_*: maximum quantum yield. (**b**) Response of bell diameter (BD) and wet weight (WW) towards bleaching (mean, n = 12). c: Visualization of photosynthetic efficiency (i.e., *F_o_*, *F_m_*, and *F_v_/F_m_*).


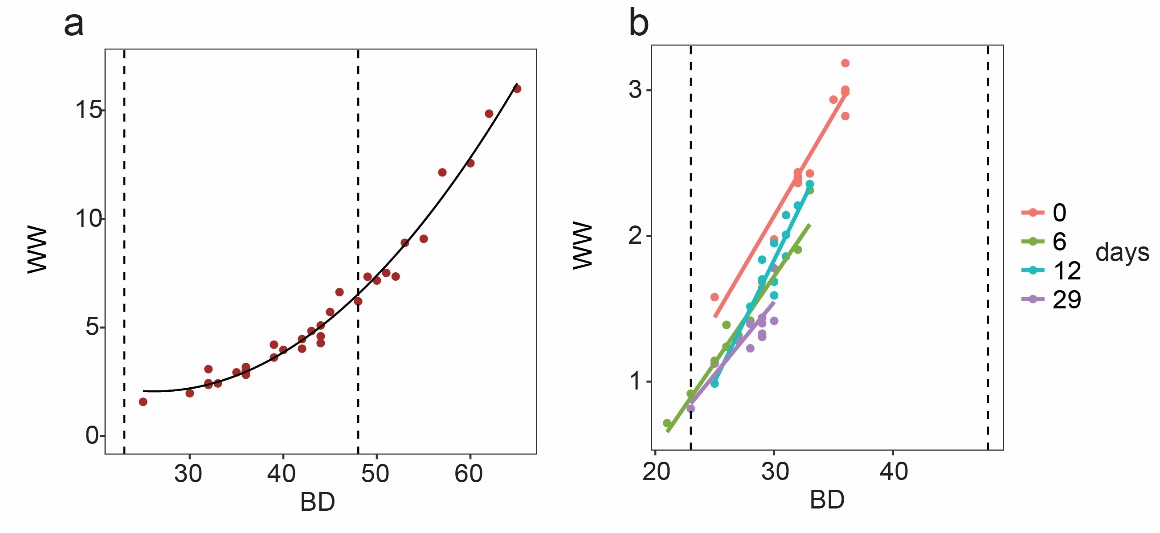


**Fig.S2. Relationship between wet weight (WW) and bell diameter (BD)** in (**a**) symbiotic (n = 35) [WW_symbiotic_ = 8.538 + (-0.494*BD) + (0.009*BD2)] and (**b**) aposymbiotic *C. xamachana* (n = 12) [WW_aposymbiotic_ = -1.440 + (0.100*BD)]. Different colors indicate the number of days after the start of the four day menthol bleaching. Dashed vertical lines indicate minimum and maximum BD of medusae employed in the pulse-chase experiment.

**
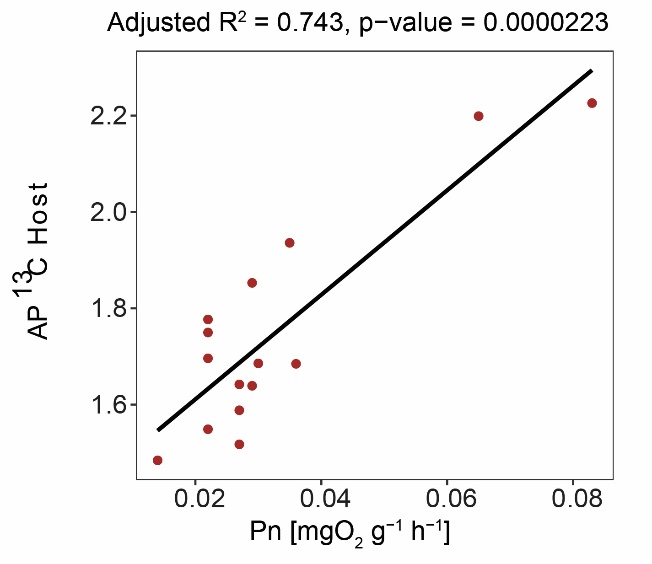
**

**Fig.S3. Net primary production (P_n_) and host carbon enrichment.** Host fraction enrichment expressed as AP^13^C light-incubated medusae (SymL) from all incubations (pulse, chase 3 h and chase 6 h; n = 15; y = 1.394 + 10.845x).
