# Supplementary material for: Holobiont nitrogen control and its potential for eutrophication resistance in an obligate photosymbiotic jellyfish
Source: Microbiome. 2021 Jun 2;9:127. doi: 10.1186/s40168-021-01075-0 (PMC8173792; doi:10.1186/s40168-021-01075-0)
Supplement: Supplementary file 3 — Additional file 2: Text S1. Extended Material and Methods. Text S2. OTU based bacterial analysis. [file 40168_2021_1075_MOESM3_ESM.docx]

**Holobiont nitrogen control and its potential for eutrophication resistance in an obligate photosymbiotic jellyfish**

Till Röthig^1,2,3,#,*^, Giulia Puntin^1,4,#^, Jane CY Wong^1^, Alfred Burian^5,6^, Wendy McLeod^1^, David M Baker^1,*^

^1^ The Swire Institute of Marine Science and School of Biological Sciences, The University of Hong Kong, Hong Kong, SAR China

^2^ Department of Bioresources, Fraunhofer Institute for Molecular Biology and Applied Ecology, Giessen, Germany

^3^ Department of Biology, University of Konstanz, Konstanz, Germany

^4^ Department of Animal Ecology & Systematics, Justus Liebig University, Giessen, Germany

^5^ Marine Ecology Department, Lurio University, Nampula, Mozambique

^6^ Department of Computational Landscape Ecology, UFZ– Helmholtz Centre for Environmental Research, Leipzig, Germany

# contributed equally

* Corresponding author

**Additional Information File 2 – Extended Material and Methods,**

**OTU based microbial analysis**

#### **Text S1: Extended Material and Methods**

#### Samples processing

Frozen *C. xamachana* samples were crushed with clean and sterile pestle and mortar and further homogenized (30 s at medium-high speed, Tissue Tearor, Biospec). Then 400 µL aliquots from the 15 individuals sampled at T1 (after pulse; treatments SymL, SymD, and ApoL) samples were taken for analyses of the associated microbial communities (see below). The tissue homogenate of each sample was then separated by centrifugation into host and algal symbiont fractions. Homogenates were centrifuged (490 × g for 5 min), the supernatant containing mostly host tissue was centrifuged again (3000 × g for 5 min) to remove the remaining algal cells (pellet discarded). The supernatant was frozen at -20°C for host SIA. The algal pellet produced in the first spin was rinsed twice by adding reverse osmosis (RO) water, and additional centrifugation (3000 × g for 5 min). To subsample for algal cell counts, the pellet was re-suspended in 2 mL, of which 100 µL were transferred into 1.5 mL 95 % ethanol, chilled at -20°C, then *Symbiodiniaceae* cells were counted using a Neubauer hemocytometer under a light microscope (Olympus Optical, mod. CHK) at 400× magnification. Four counts were averaged per individual and *Symbiodiniaceae* density was calculated as cell number per g WW of jellyfish. The remaining algal suspension was centrifuged (3134 × g for 10 min) and the algal pellet was frozen at -20°C for SIA. Prior to SIA, algal pellet and host supernatant were freeze-dried, weighted and packed into 3 × 4 mm tin capsules. To avoid cross-contamination, controls and enriched samples were processed on different days and the latter prepared and analyzed separated by tissue fraction (host<symbionts) and with predicted increasing enrichment (controls<chase6h<chase3h<pulse).

#### PCR conditions

Amplifications were performed in triplicates and adjusted to a total volume of 20 µL each containing 10 µL PCR multiplex mix (Qiagen multiplex PCR kit), 0.5 µM of each primer, 1 µL template DNA containing 10 - 80 ng DNA, and H_2_O. PCRs were set to an initial denaturation at 95°C for 15 min followed by 27 cycles each with 30 s at 95°C, 90 s at 55°C, and 30 s at 72°C. The final elongation was set at 72°C for 10 min. Successful amplifications were confirmed using 10 µl PCR products on a 2 % agarose gel, then triplicate PCRs were pooled for each sample. Samples were sent to the Centre for PanorOmic Sciences (CPOS), The University of Hong Kong, for library preparation, bead clean-up, pooling in equimolar ratios and sequencing on a MiSeq Nano v2 PE250 run with 20 % PHiX.

**Text S2: OTU based analyses**

OTU based analyses – Material and methods

To compare the bacterial communities associated with *C. xamachana* with the literature, an additionally OTU based analysis was performed. This approach mirrors well published methodology (e.g. extractions, primer, software) in the field (e.g. coral, *Exaiptasia*) thus highlighting similarities in cnidarian associated microbiomes. The analysis was conducted using mothur v.1.44 [1]. Forward and reverse reads were split according to barcodes and assembled to contigs. Contigs >310 bp and those containing ambiguous bases were discarded. Identical sequences were merged and ‘count.seqs’ was used to keep count of all sequences. Sequences were aligned against SILVA [2], quality filtered and pre-clustered (3 bp difference) [3]. Then sequences that occurred only once over all samples and chimeric sequences were excluded (VSEARCH, [4]). All remaining sequences were classified against SILVA v.138 using an 80 % bootstrap cut-off, then - if present - chloroplasts, Mitochondria, *Archaea*, Eukaryotes and unknown sequences were removed. To provide an overview, stacked column plots representing bacterial community compositions at the taxonomic level ‘family’ were constructed. Next, sequences were clustered into operational taxonomic units (OTUs) at 97 % similarity. To account for contamination, OTUs that occurred more than 10 % in controls (i.e. sum of extraction and sequencing control) compared to the sum of all other samples were removed (i.e., 9 OTUs removed, 6 occurred only in controls, 3 occurred > 10 % in controls, OTU140 (*Lawsonella* sp.) most abundant contaminant). For further analysis the control was discarded and alpha diversity indices (Chao1, Simpson evenness, and inverse Simpson index) were calculated. To compare significant differences in alpha diversity indices between sites assumptions were tested, i.e. normal distribution (Shapiro-Wilk W > 0.92, *p* > 0.1) and homogeneity (*p_Barlett&Leven_* > 0.1), prior to performing single-factor ANOVAs or, when assumptions were not met, Mann-Whitney U tests in R. All samples were then subsampled to 10,165 sequences (lowest number of sequences available for any sample) and a Bray-Curtis dissimilarity matrix was constructed in mothur. Based on this, a principal coordinate analysis (PCoA) and an Analysis of Molecular Variance (AMOVA) were calculated. The putative core microbiome was identified based on subsampled data and includes all OTUs present in all sampled medusae (n = 15), the symbiotic microbiome (symbiome) includes all OTUs present in all symbiotic medusae (n = 10) but not in all aposymbiotic samples, and the aposymbiotic microbiome (apobiome) includes all OTUs present in all aposymbiotic medusae (n = 5) but not in all symbiotic samples.

OTU based analyses – Results

We produced 16 16S rRNA gene libraries containing 252,674 contigs from one extraction control, five SymL, five SymD, and five ApoL *C. xamachana* samples. After quality control, chimera detection, and removal of undesired and rare (n = 1 over all samples) sequences, 191,141 sequences with an average length of 292 bp remained in the dataset. To assess sample and treatment specific differences in bacterial community composition, we classified all sequences to the family level (Additional Information File 2 - Fig. S4). While the control was markedly different from all other samples, medusae showed little variation and were dominated by *Moraxellaceae* (>60 %) and *Pseudomonadaceae* (~15-25 %).


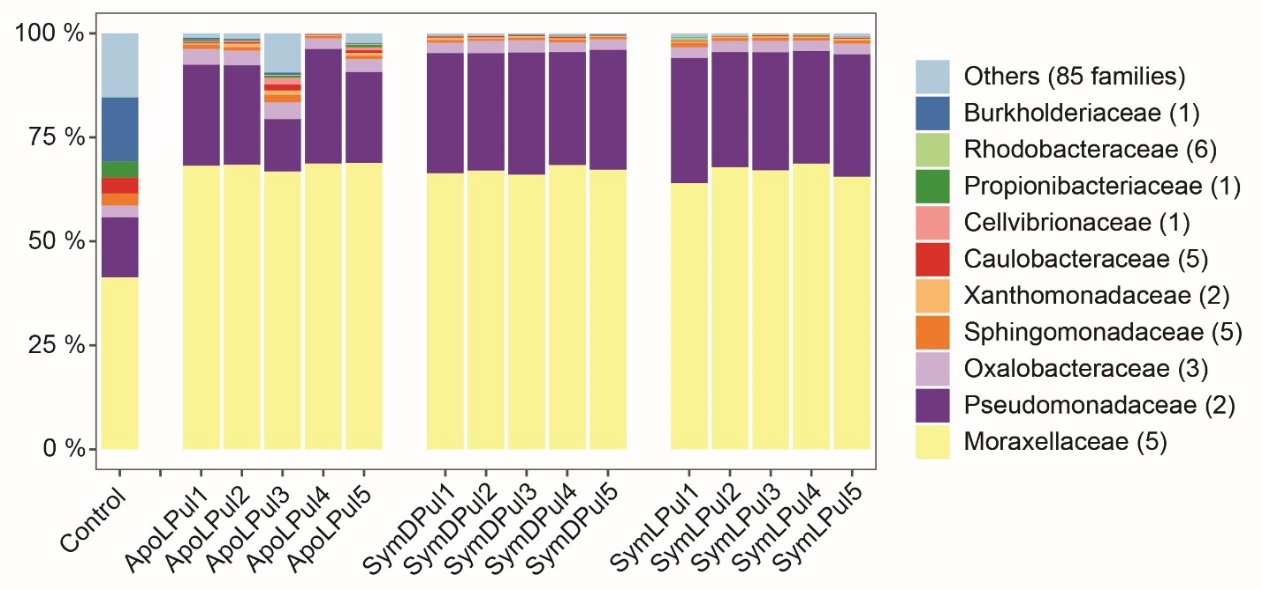


**Figure S4**. Stacked column plot representing the bacterial community composition associated with *C. xamachana* on the phylogenetic level family (SILVA database, bootstrap ≥80). Each color represents one of the 10 most abundant families, all 85 other taxa are grouped as ‘others’.

Numbers in parenthesis depict daughter genera present in each family. Treatments: Apo = aposymbiotic, Sym = symbiotic, L = light, D = dark, Pul = sampled after pulse incubation.

To assess differences between each treatment we clustered sequence data to operational taxonomic units (OTU; 97 % similarity) after removal of contaminants and the control. The assembled rarefaction curves indicated that with >10,000 sequences in each sample the majority of bacterial diversity is represented (Additional Information File 2 - Fig. S5). Number of observed species and Chao1 estimator of species richness were about twice as large in the aposymbiotic samples than in both symbiotic groups (i.e. light and dark), while the trend was inverted for Simpson evenness (Additional Information File 2 - Table S1). Biodiversity measured as inverse Simpson Index was similar for all three treatments. Comparison between aposymbiotic and symbiotic medusae were not significant for Chao 1 (*p_Mann-Whitney_*=0.055), Simpson evenness (*p_Mann-Whitney_*=0.075), and inverse Simpson (*p_Mann-Whitney_*=0.389) albeit the low *p*-value for species richness indicates a trend for richer communities in the aposymbiotic specimens.


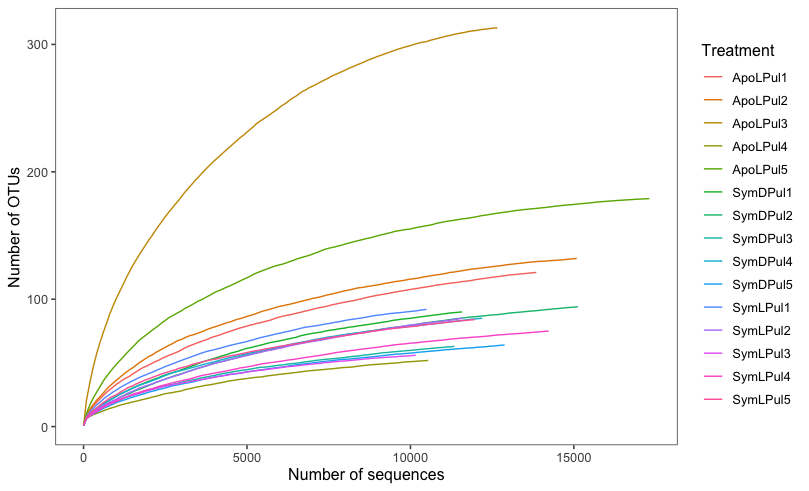


**Figure S5.** Rarefaction curves illustrating OTU richness as a function of sampling effort.

**Table S1.** Summary statistics detailing microbial communities associated with *C. xamachana.*

|  | **# of sequences** | **observed OTUs** | **Chao 1** | **Simpson evenness** | **inverse Simpson** |
| --- | --- | --- | --- | --- | --- |
| Control | 87 | 15 | 16.2 | 0.271 | 4.06 |
| ApoLPul1 | 13851 | 121 | 138.1 | 0.017 | 2.08 |
| ApoLPul2 | 15087 | 132 | 140.6 | 0.016 | 2.06 |
| ApoLPul3 | 12662 | 313 | 317.7 | 0.007 | 2.34 |
| ApoLPul4 | 10545 | 52 | 69.5 | 0.039 | 2.01 |
| ApoLPul5 | 17310 | 179 | 185.3 | 0.011 | 2.03 |
| **Mean** | **13891** | **159** | **170.2** | **0.018** | **2.10** |
| **STDV** | **2541** | **97** | **92.2** | **0.012** | **0.14** |
| SymDPul1 | 11579 | 90 | 105.0 | 0.023 | 2.10 |
| SymDPul2 | 15123 | 94 | 111.1 | 0.022 | 2.08 |
| SymDPul3 | 11345 | 63 | 84.2 | 0.034 | 2.12 |
| SymDPul4 | 12195 | 85 | 100.6 | 0.024 | 2.04 |
| SymDPul5 | 12881 | 64 | 83.7 | 0.032 | 2.08 |
| **Mean** | **12625** | **79** | **96.9** | **0.027** | **2.08** |
| **STDV** | **1518** | **15** | **12.4** | **0.005** | **0.03** |
| SymLPul1 | 10490 | 92 | 105.1 | 0.024 | 2.21 |
| SymLPul2 | 11599 | 85 | 114.0 | 0.024 | 2.07 |
| SymLPul3 | 10165 | 56 | 64.5 | 0.038 | 2.10 |
| SymLPul4 | 14230 | 75 | 87.5 | 0.027 | 2.01 |
| SymLPul5 | 11967 | 84 | 97.5 | 0.025 | 2.13 |
| **Mean** | **11690** | **78** | **93.7** | **0.028** | **2.11** |
| **STDV** | **1605** | **14** | **19.0** | **0.006** | **0.07** |

Treatments: Apo = aposymbiotic, Sym = symbiotic, L = light, D = dark, Pul = sampled after pulse incubation.

#### Distinct bacterial communities associated with symbiotic and aposymbiotic C. xamachana

To identify differences between treatments and/or symbiotic states, we compared bacterial community profiles over medusae and treatments in a PCoA based on a Bray-Curtis dissimilarity matrix (Additional Information File 2 - Fig. S6). ApoL samples were comparably broadly dispersed and clustered away from SymL and SymD samples, which were clustering relatively close together. These finding were corroborated by an Analysis of Molecular Variance (AMOVA), showing significant differences between ApoL and SymL (*p_AMOVA_*=0.013), ApoL and SymD (*p_AMOVA_*=0.013), but not between SymL and SymD (*p_AMOVA_*=0.718). The similarities between light and dark treatments for symbiotic medusae including alpha diversity indices, PCoA, and AMOVA suggest that the 5h incubation did not affect the associated bacterial communities.


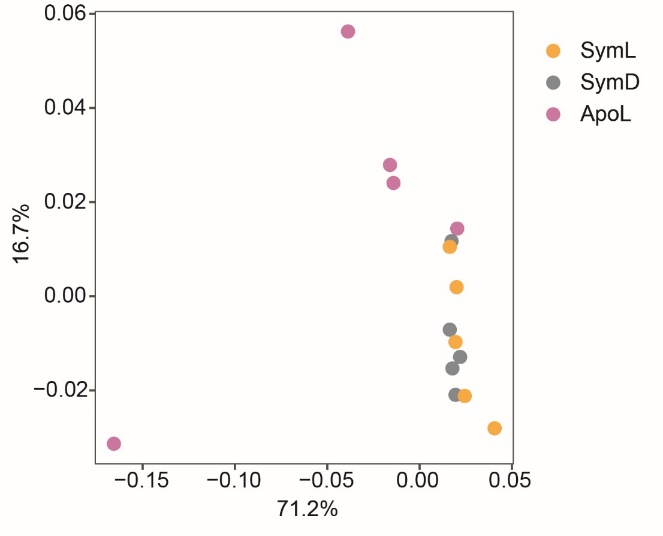


**Figure S6.** Clustering of *C. xamachana* samples based on Bray-Curtis dissimilarity of microbial community abundances in a principal coordinate analysis (PCoA) (R^2^ = 0.99).

Treatments: Apo = aposymbiotic, Sym = symbiotic, L = light, D = dark, Pul = sampled after pulse incubation. Percentage values at each axis detail its explanatory power.

To identify OTUs that – based on their ubiquity – may be of functional importance [5], we firstly pooled all symbiotic samples (i.e. SymL and SymD). Next, we characterized the core microbiome of all jellyfish, the symbiome, and the apobiome. The core microbiome, i.e. the OTUs present in all 15 medusae, consisted of 18 OTUs, including 13 of the 15 most abundant taxa (Additional File 3: Table S6). Curiously, the symbiome consisted only of OTU0017 (unclassified *Rhodobacteraceae*), which also occurred in one aposymbiotic sample at a low abundance (n = 4). The apobiome consisted of 9 OTUs and its most abundant member was OTU009 (*Sphingomonas* sp.).

**References for Additional Information File 2**

1. Schloss PD, Westcott SL, Ryabin T, Hall JR, Hartmann M, Hollister EB, et al. Introducing mothur: Open-Source, Platform-Independent, Community-Supported Software for Describing and Comparing Microbial Communities. Appl Environ Microbiol. 2009;75:7537–41. https://doi.org/10.1128/aem.01541-09 American Society for Microbiology.

2. Pruesse E, Quast C, Knittel K, Fuchs BM, Ludwig W, Peplies J, et al. SILVA: a comprehensive online resource for quality checked and aligned ribosomal RNA sequence data compatible with ARB. Nucleic Acids Res. 2007;35:7188–96. https://doi.org/10.1093%2Fnar%2Fgkm864 Oxford Academic.

3. Huse SM, Welch DM, Morrison HG, Sogin ML. Ironing out the wrinkles in the rare biosphere through improved OTU clustering. Environ Microbiol. 2010;12:1889–98. http://dx.doi.org/10.1111/j.1462-2920.2010.02193.x American Society for Microbiology

4. Rognes T, Flouri T, Nichols B, Quince C, Mahé F. VSEARCH: A versatile open source tool for metagenomics. PeerJ.2016 https://doi.org/10.7717/peerj.2584 PeerJ Inc.

5. Ainsworth T, Krause L, Bridge T, Torda G, Raina J-B, Zakrzewski M, et al. The coral core microbiome identifies rare bacterial taxa as ubiquitous endosymbionts. ISME J. 2015;9:2261–74. http://dx.doi.org/10.1038/ismej.2015.39 Nature Publishing Group
